# The 1918 influenza pandemic in Montevideo: The southernmost capital city in the Americas

**DOI:** 10.1111/irv.12619

**Published:** 2019-02-14

**Authors:** Juan Cristina, Raquel Pollero, Adela Pellegrino

**Affiliations:** ^1^ Laboratorio de Virologia Molecular Centro de Investigaciones Nucleares Facultad de Ciencias Universidad de la Republica Montevideo Uruguay; ^2^ Facultad de Ciencias Sociales Universidad de la República Montevideo Uruguay

**Keywords:** 1918, influenza, mortality, pandemic, temperate

## Abstract

**Background:**

Few studies have addressed the impact and dynamics of the 1918‐1919 influenza pandemic in temperate regions of South America.

**Objective:**

To identify key factors for influenza onset, spread, and mortality in Montevideo and Uruguay in 1918‐1919.

**Methods:**

An analysis of official national records of the public health system of Uruguay was performed.

**Results:**

From November to December of 1918 (spring), a total of 131 deaths due to influenza occurred in Montevideo and a total of 296 deaths accounted from July to September of 1919 (winter) in the same city. The total deaths attributed to influenza in Uruguay in 1918 and 1919 were 926 and 1089, respectively. In contrast, the mean annual mortality attributed to influenza in Uruguay from 1908 to 1917 was 50.9. A pattern of age‐shift in mortality in the two pandemic waves studied was observed.

**Conclusions:**

The results of studies revealed that Montevideo was first hit by the devastating second wave of the pandemic of 1918, arriving Montevideo at the end of the spring of that year. The third wave arrived by July 1919, in the winter season, and in the capital city was as severe as the second one.

## 1 INTRODUCTION

The 1918‐1919 influenza pandemic was the deadliest in history, with estimates of global mortality ranging from 20 to 100 million people.^1^ Previous studies revealed that the pandemic first manifested through a mild wave (with some exceptions) mainly in the Northern Hemisphere^2^, although other places in the Southern Hemisphere, such as Singapore, Australia, and New Zealand, were affected between March and July of 1918.^3^ Then, influenza virus spread globally with a devastating second wave, which lasted from approximately August 1918 to early 1919, and a third wave that occurred between February and April 1919 was intermediate in severity.^4^

Important factors about the pandemic, especially its transmission dynamics and the mechanisms for its extreme virulence, still need further studies.^5^

Previous reports have revealed characteristic features of the 1918‐1919 influenza pandemic, including increased mortality rates in young adults, relative to seasonal epidemics, and the occurrence of multiple pandemic waves over short periods of time.^1^, ^6^ Unfortunately, the dynamics and impact of the 1918‐1919 pandemic are poorly understood in temperate subtropical regions of the southern cone of South America. A better understanding of these patterns is essential to better prepare for future influenza pandemics.^6^ In order to gain insight into these matters, we analyzed official national records of the public health system of Uruguay.

## 2 METHODS

### 2.1 Sources of data

The series used for this study derive from official published statistical series for deaths by age, sex, and cause of death (Instituto Nacional de Estadística—Ministerio de Salud Pública). Causes were grouped together according to the International Classification of Diseases adopted by the country in 1901 (ICD‐1). We also used official Montevideo's series of deaths (Dirección de Censo y Estadística, Intendencia Municipal de Montevideo). To examine morbidity and mortality in the 1918‐1919 pandemic, we also used relevant information from Boletín del Consejo Nacional de Higiene (years 1918, 1919 and 1920). We considered all records available from January 1918 to December 1919. Individual entries containing age, sex, date, and cause of death from all records were tabulated.

As denominator of mortality rates, we have referenced two population projections: one for Uruguay^7^ and another for Montevideo.^8^

### 2.2 Estimation of transmission characteristics (reproduction number)

The basic reproduction number (*R*_0_) is defined as the average number of secondary cases generated by a primary case at the onset of an epidemic in an entirely susceptible population^9^. A related quantity is the reproduction number, *R*, which captures partial immunity in the population due to previous exposure of the population to related influenza viruses or vaccination campaigns.^10^ We estimated *R* for the 1918 and 1919 pandemic virus in Uruguay by using method that relies on the epidemic growth rate, a measure of how fast the number of cases increases over time.^11^ Because of the uncertainty associated with duration of the latency and infectious periods for influenza, we considered periods of 1.5 and 3 days, respectively.^12^, ^13^ We estimated *R* using the number of cases of influenza per day attended at the National Public Assistance (Asistencia Pública Nacional) from October 19th to October 31st for the pandemic wave of 1918 and from July 1st to July 23rd for the pandemic wave of 1919.

### 2.3 Pandemic mortality burden

In order to gain insight into the 1918 pandemic mortality burden in our country, we considered the available records of respiratory deaths for Montevideo for the period 1912‐1919. We considered all causes of respiratory death for Montevideo, which includes the sum of respiratory death caused by influenza, laryngeal tuberculosis, pulmonary tuberculosis, acute bronchitis, bronchopneumonia, pneumonia, pleurisy, and gangrene of the lung. Pandemic mortality burden in was calculated from the excess of respiratory deaths for the pandemic years 1918 or 1919 by subtracting the absolute number of respiratory deaths during these years from the average of respiratory deaths during pre‐pandemic years represented in the dataset (1912‐1915). We did not include 1916 in the dataset due to the fact that this year was also particularly different (see below). Besides, the mortality rate by influenza per 100 000 inhabitants in Uruguay was calculated for the period 1908‐1919 and compared with rates obtained from other documented causes of death in the country (circulatory diseases, cancer, pulmonary tuberculosis, and accidents).

## 3 RESULTS

### 3.1 The historical context

**Figure 1:**
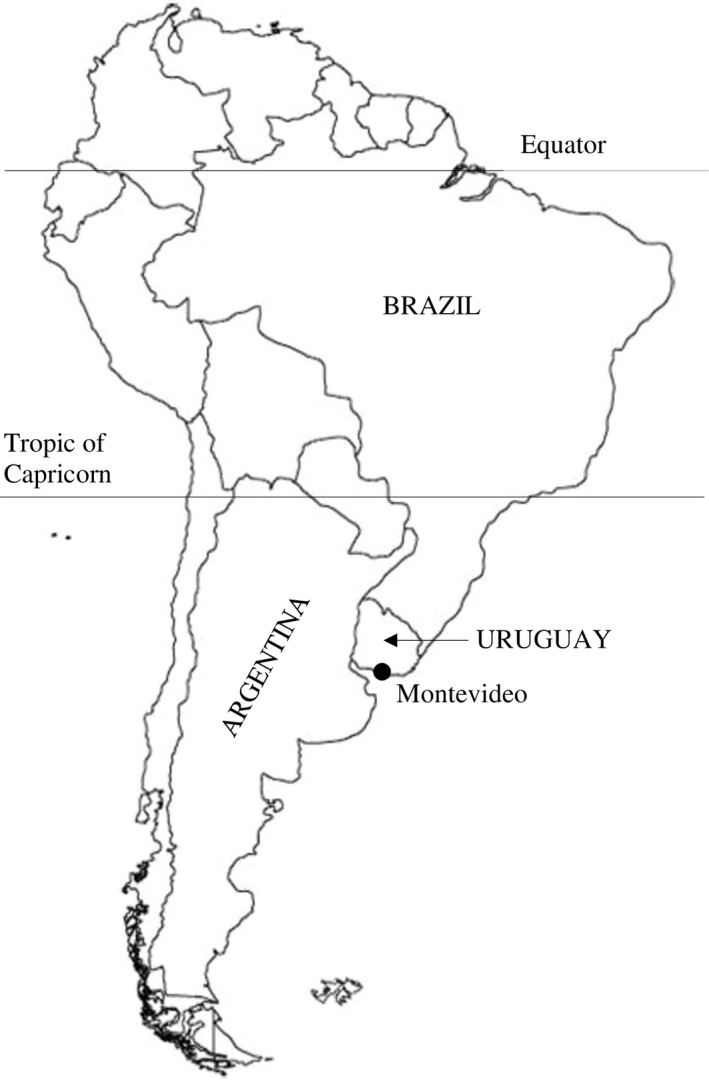
Current political map of South America. The figure shows the location of the study (Uruguay) and its capital Montevideo

Montevideo, Uruguay's capital city, is considered the most southern capital of the Americas. Located in a coastal region by the Rio de la Plata (latitude: −34.83346, longitude −56.16735; see Figure 1), Montevideo enjoys a well‐defined four‐season year, with average temperatures ranging from 22.6°C in summer (January) to 10.6°C in winter (July) and year‐round average precipitations ranging from 67 to 88 mm.^14^

By 1900, life expectancy at birth was around 48 years and infant mortality rate was a little <100 per every 1000 live births.^15^, ^16^ When the influenza pandemic reached Uruguay in 1918, the healthcare indicators were satisfactory for the standards of this time and close to those reached by developed countries. The impact of the influenza pandemic alarmed the Uruguayan society, and the number of cases was significant for the country's small population.

### 3.2 Morbidity and reproduction number estimates

**Figure 2:**
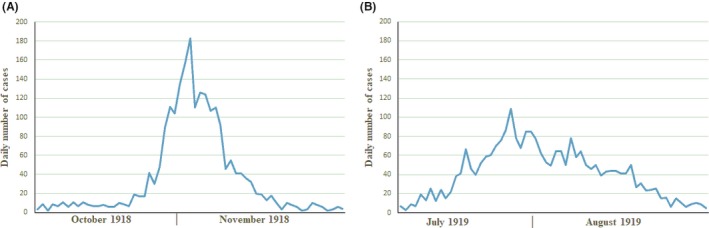
Morbidity of influenza in Uruguay at peak pandemic months in 1918 and 1919. The daily number of influenza patients attended at Servicio de Primeros Auxilios, Asistencia Pública Nacional per day is shown

In order to gain insight into the spread and the morbidity caused by pandemic waves in 1918 and 1919 in Uruguay, we analyzed the daily number of cases of influenza at peak pandemic months, being October and November for 1918 and July and August for 1919 influenza waves. The results of these studies are shown in Figure 2.

A sharp increase in the number of cases was observed by late October and the beginning of November of 1918, when the pandemic hits Uruguay for the first time. Interesting, this hit took place in the late spring of the Southern Hemisphere, far away from the expected influenza season. On the other hand, the pandemic hits Uruguay for the second time during winter 1919, with a peak in late July, in the expected influenza season (see Figure 2). Then, in order to study the epidemic spread and growth in the Uruguayan population and to compare these results with similar studies performed in our region and elsewhere, we estimated the reproduction number (*R*) for the 1918 and 1919 pandemic virus in Uruguay^10^. The *R* for the 1918 and 1919 influenza pandemic in Uruguay was estimated to be 2.0 and 2.2, respectively, assuming a short generation interval of 3 days. Although *R* estimates were 1.3‐1.8 that were found in most locations in the Americas, assuming a 3‐day generation interval, *R* values of 2.0‐2.5 have been also observed.^10^ If a longer interval of 4 days is considered, *R* estimations of 1.9 and 2.0 were obtained for 1918 and 1919, respectively.

### 3.3 Comparison of morbidity and mortality due to influenza in pre‐pandemic and pandemic years in Uruguay

**Figure 3:**
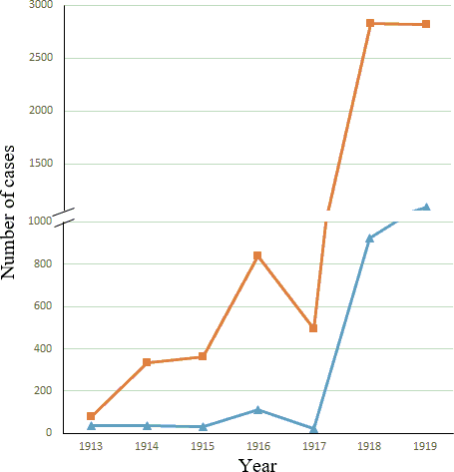
Morbidity and mortality due to influenza from 1913 through 1919 in Uruguay by year. The total number of death due to influenza is shown for each year. Morbidity is shown in orange and marked with squares, while mortality is shown in blue and marked with triangles

In order to compare the morbidity and mortality due to influenza in pre‐pandemic and pandemic years in Uruguay, we compared the yearly records obtained on morbidity and mortality due to influenza from 1913 through 1919. The results of these studies are shown in Figure 3.

Pre‐pandemic years are characterized by low mortality figures. Interestingly, a peak on morbidity and mortality is observed in 1916. A sharp increase in morbidity and mortality is observed in the pandemic years (see Figure 3).

### 3.4 Pandemic mortality burden

The annual respiratory deaths in Montevideo for 1918 and 1919 were 2115 and 2403, respectively. When these figures were compared to the calculated base respiratory mortality burden in Montevideo (mean of respiratory deaths for the pre‐pandemic years 1912‐1915), they represent an excess of respiratory deaths of 513 and 801 for that years, respectively, which represent 0.11% and 0.16% of the total population of the city on that years. Interestingly, an excess of respiratory deaths of 585 was also observed in 1916 (0.13% of the city population).

**Figure 4:**
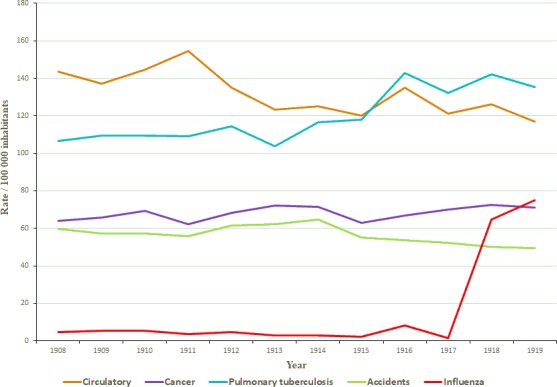
Rate of influenza deaths in Uruguay per 100 00 inhabitants for the period 1908‐1919. The rate of deaths due to circulatory system failure, cancer, pulmonary tuberculosis, accidents, and influenza for each year is shown

In order to gain insight into the evolution of influenza mortality rate in Uruguay (1908‐1919), we compared the influenza mortality rates per 100 000 inhabitants with other causes of death for the same time period. The results of these studies are shown in Figure 4. An increase in pulmonary tuberculosis rate is observed after 1916. Importantly, a sharp increase in the rate obtained for influenza is clearly observed for 1918 and 1919 years, roughly matching cancer mortality rate and being higher than the external causes mortality rate in those years.

**Figure 5:**
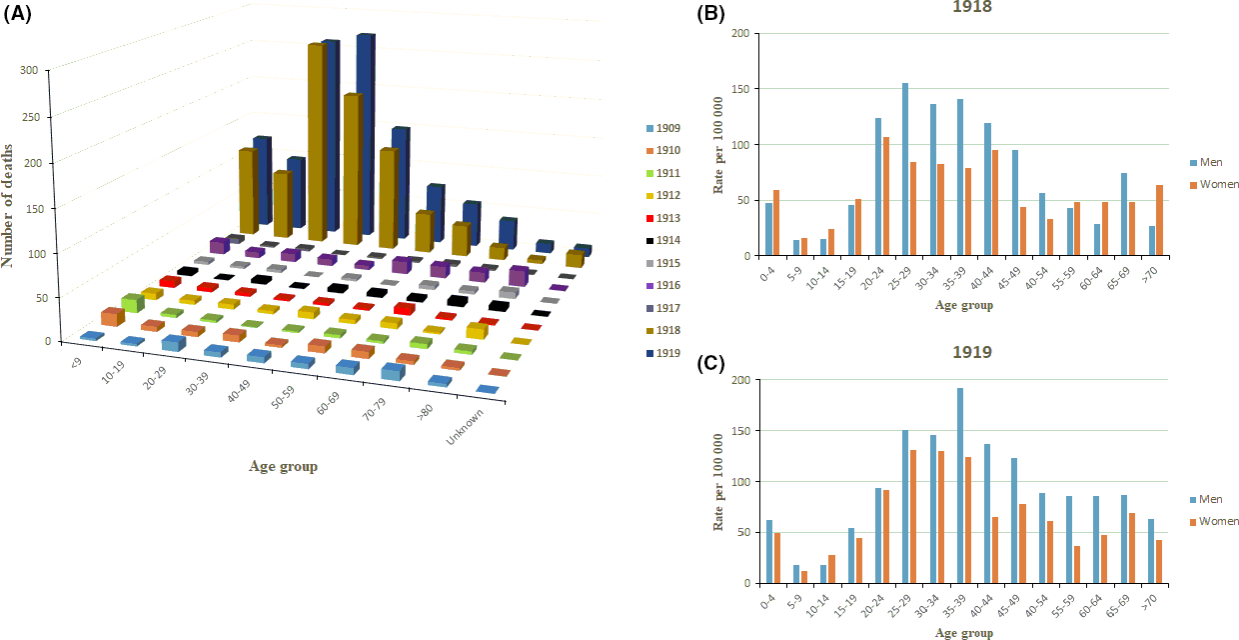
Deaths due to influenza in Uruguay for the period 1909‐1919. In (A), the total number of deaths by year due to influenza divided by age‐groups is shown. In (B) and (C), the death rates of influenza per 100 000 inhabitants divided by age‐groups and sex for 1918 and 1919, respectively, are shown

Then, the incidence of death due to influenza per age‐group was studied for the period 1909‐1919 in Uruguay. The results of these studies are shown in Figure 5.

As it can be seen in the figure, in absolute numbers, the age‐group most heavily affected in pandemic years was young adults (29‐49 years old), followed by children <9 years old (ie, the same incidence of death due to influenza in children <9 years old equals the total death toll due to influenza in 1916; see Figure 5A). Seniors >70 years old were not significantly affected in comparison with the other age‐groups studied (Figure 5A). Mortality rates by age‐group and sex for pandemic years 1918 and 1919 show that adult men (age‐groups: 25‐49) were the most affected. Among children, the highest mortality is observed in group aged 0‐4 (see Figure 5B,C).

## 4 DISCUSSION

The 1918 influenza pandemic spread all over the entire world and killed tens of millions of people.^17^ The exact origin of the 1918 influenza pandemic still remains to be determined^18^, ^19^. The causes for its transmissibility, virulence, and unique age pattern remain to be clearly established.^20^ Analysis of viral RNA recovered from preserved lung tissue samples has confirmed the presence of an influenza A/H1N1 virus in the pandemic wave in the Northern Hemisphere in the autumn of 1918 and its recrudescence in winter of 1919.^21^ Previous reports have revealed that the winter months of 1915 and 1916 showed an increased influenza activity and higher records of deaths due to influenza in the United Kingdom^22^ and the USA.^18^ Whether these outbreaks or the ones reported in Europe in 1916 and 1917 were caused by a virus related to the pandemic one remains unknown. Interestingly, we observed an increased morbidity and mortality in Uruguay in the winter of 1916 (see Figure 3), a winter particularly cold in our country. Moreover, an increase in respiratory mortality deaths due to pneumonia and bronchopneumonia was also observed in Montevideo during that year. More studies will be needed in order to address these facts.

Quantitative analyses of age‐specific death rates, transmissibility, and dissemination patterns of the 1918 influenza pandemic have been performed in the USA,^18^ Europe,^2^, ^23^ Taiwan,^24^ and Singapore.^25^ In the case of Latin America, studies have been done in Mexico,^26^ Brasil,^27^ Peru,^1^ Colombia,^10^ and Chile.^28^ These studies revealed the pandemic's unusual severity in young adults, occurrence in multiple waves, and higher transmission potential than that of seasonal epidemics^27^ in agreement with the results of this work.

The results of these studies revealed that the most affected age‐groups of the pandemic of 1918 were young adults, ranging from 20 to 49 years old, and adults up to age‐group 45‐49, followed by young children <5 years old (see Figure 5A‐C). This age‐shift in mortality toward young adults is in agreement with previous reports on the global assessments of the 1918 pandemic.^29^, ^30^ Interestingly, when pandemic years are divided by age‐group and sex, it is possible to observe that adult men were the most affected groups (see Figure 5B,C). Nevertheless, in contrast to previous studies done in the Latin American region,^10^ the elderly population in Uruguay (>60 years old) resulted to be protected from influenza‐associated death in 1918, by comparison with all other age‐group, as previously observed in the USA^18^ and Europe.^31^ Previous studies have hypothesized that childhood exposure to influenza viruses before 1870 might account for prior immunity among elderly persons during the 1918 pandemic. This is in line to findings by studies on the pandemic of influenza A H1N1 in 2009, which show that the risk of death was lower during the 2009 pandemic than during seasonal epidemics for persons older than 60 years of age.^32^

In these studies, the *R* estimations for the 1918 and 1919 influenza pandemic in Uruguay were 2.0 and 2.2, respectively, assuming a 3‐day generation interval. Transmissibility estimates for the 1918‐1920 pandemic period are in the range of 1.5‐5.4 for community‐based settings in different regions of the world.^33^, ^34^ Comparable transmissibility estimates in other locations in Latin America, such as Peru,^1^ Colombia,^10^ and Mexico city,^26^ are in the range of 1.3‐1.8. Nevertheless, high *R* estimations have been found in Toluca (México) with values ranging from 2.0 to 2.5.^27^ These findings reveal differences in the region regarding transmissibility of influenza. More studies will be needed to establish if these differences in reproduction number estimates across the region reflect differences in attack rates or there are local factors affecting influenza transmission^35^.

The analysis of the 1918‐1919 influenza pandemic in Uruguay revealed a unique epidemiological pattern by comparison with the expected one, revealing a single wave of influenza for 1918, shifted to the end of spring (November, 1918; Figure 2). We did not identify an earlier wave for that year, revealing that it is likely that the virus had not been introduced to the country in the earlier months or the winter of 1918. For these reasons, we believe that the second pandemic wave of the pandemic of 1918 was the one that first hit Uruguay toward the end of that year. A similar shift of seasonality was also observed in the South of Brazil.^27^ Interestingly, a third wave of influenza hits Uruguay in the winter of 1919 (Figure 2). Post‐pandemic waves have been observed for 1919‐1920 in other regions of the world.^18^

Measuring the burden of historical influenza pandemics is not an easy task and may be based on suboptimal mortality data.^36^ When using annual all‐cause mortality data, a ∼40‐fold between‐country variation in pandemic mortality burden has been found.^29^ Recent studies revealed that a more precise pandemic burden estimate can be found from focusing the analysis of annual respiratory deaths rather than all causes of death.^36^ Analysis of annual respiratory deaths in Montevideo revealed a pandemic mortality burden of 0.11% and 0.16% for 1918 and 1919, respectively. These figures are comparable to the results found by similar studies in Denmark (∼0.2%),^29^, ^36^ and studies performed in England and Wales^37^ revealed that the first wave of the pandemic, interestingly occurring in summer, outside the typical influenza season, had very low figures of pandemic mortality burden (0.03%), while the second and third waves revealed figures of 0.27% and 0.10%. Nevertheless, all these figures are far below the mortality rates estimated for other countries, such as Kenya (4.0%‐5.8%) or India (up to 8% in one Indian province)^29^, although a different methodology has been used in the studies carried out in these countries.

According to Del Panta & Livi Bacci^38^, a 50% or more increase in mortality may endanger demographic equilibrium and can be classified as a crisis. Previous studies performed at Montevideo revealed that the last mortality crisis coincides with the cholera epidemic in 1868 (71.3% above the level of normal mortality). The previous crisis was in 1857, year of the yellow fever epidemic (56.3% above the normal level).^39^

The death values for years 1918 and 1919 are very far from being considered a crisis capable to affect demographic equilibrium in Uruguay. This is important to contextualize these years mortality rise, although important, with respect to what is regarded as a mortality crisis.

## 5 CONCLUSIONS

The results of these studies revealed that Uruguay was first hit by the devastating second wave of the pandemic of 1918, arriving at the end of the spring of that year. The third wave arrived by July 1919, in the winter season, as was as severe as the second one. The most affected age‐groups of the pandemic of 1918 in Uruguay were young adults. When mortality in pandemic years is divided by age‐group and sex, the most affected group were men adults. The elderly population (>60 years old) resulted to be significantly protected from influenza‐associated death in 1918, by comparison with all other age‐group. High *R* estimations have been found for the pandemic of 1918 in Uruguay. The analysis of the 1918‐1919 influenza pandemic in Uruguay revealed a single wave of influenza for 1918, shifted to the end of spring (November, 1918). Mortality burden in Montevideo, although important, with an excess of respiratory deaths of 513 and 801 for 1918 and 1919, respectively, (which represent 0.11% and 0.16% of the total population of the city on that years) cannot be considered a mortality crisis. The social alarm caused by influenza epidemic was probably more related to its morbidity than to the impact of its lethality.

## CONFLICT OF INTEREST

All authors declare that they have no conflict of interest.

## AUTHORS’ CONTRIBUTIONS

Juan Cristina, Raquel Pollero, and Adela Pellegrino conceived the study and design. Juan Cristina, Raquel Pollero, and Adela Pellegrino were involved in the acquisition, analysis, and interpretation of data. Juan Cristina drafted the manuscript. Raquel Pollero and Adela Pellegrino were involved in the critical revision of the manuscript for important intellectual content.

## ETHICS

The historical records reported in this work are publicly available. Data were processed according to Uruguayan Law No. 18.331 on protection of personal data.
